# Consistency of linkage results across exams and methods in the Framingham Heart Study

**DOI:** 10.1186/1471-2156-4-S1-S30

**Published:** 2003-12-31

**Authors:** Larry D Atwood, Nancy L Heard-Costa, L Adrienne Cupples, Daniel Levy

**Affiliations:** 1 Department of Neurology, Boston University School of Medicine, 715 Albany Street, Boston, Massachusetts, USA; 2 Department of Biostatistics, Boston University School of Medicine, 715 Albany Street, Boston, Massachusetts, USA; 3 Framingham Heart Study, National Heart Lung and Blood Institute, Boston, Massachusetts, USA

## Abstract

**Background:**

The repeated measures in the Framingham Heart Study in the Genetic Analysis Workshop 13 data set allow us to test for consistency of linkage results within a study across time. We compared regression-based linkage to variance components linkage across time for six quantitative traits in the real data.

**Results:**

The variance components approach found 11 significant linkages, the regression-based approach found 4. There was only one region that overlapped. Consistency between exams generally decreased as the time interval between exams increased. The regression-based approach showed higher consistency in linkage results across exams.

**Conclusion:**

The low consistency between exams and between methods may help explain the lack of replication between studies in this field.

## Background

The general lack of replication of genome scan results across data sets is an ongoing concern in statistical genetics [1]. Consistency (or lack thereof) within a single study with longitudinal data could provide insight into the problem of replication. The repeated measures in the Framingham Heart Study in the Genetic Analysis Workshop 13 (GAW13) data set allow us to test for consistency of linkage results within a study across time. We believe that advances in simulation have not reached the point that they adequately capture the underlying complexity that contributes strongly to consistency issues. We therefore chose to use the real data.

A new regression-based method of linkage analysis that works on arbitrary pedigrees has recently been published [2] and implemented in the publicly available MERLIN software package [3]. This method promises to be robust with respect to departures from normality, a problem that has plagued variance components linkage analysis [4]. We used MERLIN to perform linkage analysis using both variance components (VC) and regression-based linkage analysis on seven quantitative traits in the real data from the Framingham Heart Study. For six of these traits, multiple observations (three or four for each trait) were available across time for all family participants. We performed linkage analysis using both methods on each of these time points. Thus, we performed 23 genome scans using each method (46 total) in MERLIN. This allowed us to compare results across methods and across exams. To validate the MERLIN VC we also performed genome scans on the 23 quantitative traits using GENEHUNTER [5] to compare the VC implementations.

## Methods

The traits we analyzed were body mass index (BMI), total cholesterol (CHOL), glucose (GLU), high-density lipoprotein cholesterol (HDL), height (HGT), systolic blood pressure (SBP), and log-transformed triglycerides (LGTG). The effects of sex, age, age^2^, and cohort status were included as covariates in all genome scans. In order to test the robustness of regression-based linkage, we did not transform any of the variables to achieve normality (except triglycerides). To maintain as much consistency across data sets as possible, we did not correct for hypertension, diabetes, or any medication.

The 330 families were trimmed using the PEDSYS PEDTRIM program. This program removes individuals from a pedigree who do not contribute any phenotypic or genotypic information. Using the criterion of at least one marker on chromosome 22, PEDTRIM reduced the family set from 4692 individuals in 330 families to 2604 individuals in 334 families (trimming caused four families to each be split into two).

The complexity of a family is measured by the formula 2N-F, where N is the number of nonfounders and F is the number of founders. Our prior experience with GENEHUNTER indicates that a practical upper limit for the complexity of a family is 21. After trimming, there were still nine families with complexity greater than 21 (range 23–46). These nine families were cut into 19 separate pedigrees, resulting in 2588 individuals in 344 families. All analyses described in this report used these trimmed and cut family structures.

We constructed four data sets (denoted A-D) by filling out the families with data corresponding to the first four Cohort 2 exams and include individuals from Cohort 1 exams that overlapped in time with the four Cohort 2 exams, thus yielding four data sets, each with paired contemporaneous data for Cohorts 1 and 2. These four "cross-sectional" data sets represent four time points spanning approximately 16 years. For data set A, the variables were taken from Cohort 2 Exam 1 and Cohort 1 Exam 12, depending on the cohort of the individual. Cohort 2 Exam 1 and Cohort 1 Exam 12 overlap in time (1970–1971) and are thus the natural choice to achieve a cross-sectional data set of these families. Similarly, for data sets B through D, we combined Cohort 2 Exam 2 and Cohort 1 Exam 16 (1978–1979), Cohort 2 Exam 3 and Cohort 1 Exam 18 (1982–1983), and Cohort 2 Exam 4 and Cohort 1 Exam 20 (1986–1987), respectively. There were several exceptions to this scheme. There were no Cohort 1 Exams 16 or 18 for cholesterol and HDL, so data set C was not constructed for either trait and data set B used data from Cohort 1 Exam 15 rather than 16. Similarly, height was not available from Cohort 1 Exam 12, so both height and BMI data sets A were constructed using data from Cohort 1 Exam 10.

We performed VC linkage analysis on all 23 traits using GENEHUNTER and MERLIN. Both programs computed multipoint identity-by-descent (IBD) probabilities at each marker and at four equally spaced loci between each pair of markers. We also performed regression-based linkage analysis [2] on all 23 traits using MERLIN.

## Results

MERLIN computed IBD probabilities much faster than GENEHUNTER (approximately 4.5 times faster on our Pentium 4 computer). A full genome scan (including IBD calculations) on MERLIN took approximately 4.5 hours for VC linkage and 41 hours for regression-based linkage. In addition, MERLIN was limited to markers with less than 32 alleles (on our 32-bit system). One marker had 39 alleles; it was downcoded to 30 alleles.

Table 1 shows basic descriptive statistics for all the quantitative traits over all available data sets. The last letter in each trait name refers to the particular data set (A-D). The Framingham Heart Study can be considered a population-based sample, so the mean, variance (SD^2^), and heritability in this table were used as input to the regression-based linkage analyses. Heritability was computed by MERLIN as the ratio of additive polygenic variance to the total variance.

Table 2 shows all maximum LOD scores greater than 1.44 (*p *< 0.005) for VC and regression. For every LOD score greater than 1.44, the LOD score at the same point using the other method is included for comparison. For each trait, the LOD scores are ordered by chromosome, centimorgan location, and data set, in that order. There were 15 distinct regions for which one or the other method found a LOD score greater than 3.0 in at least one exam. VC analysis found 11 regions, regression found 4 regions, and there was one region in which both methods found significant linkage (chromosome 7 for cholesterol). Of the 11 significant regions from VC, 8 had some supporting evidence for linkage (LOD score > 1.0) from regression. Of the 4 significant regions from regression, 2 showed supporting evidence from VC. Perhaps surprisingly, of the 5 significant regions for which the other method did not provide some support, 4 were for height. The last line of Table 2 shows for each trait the LOD score correlation between the two methods. The correlation calculation used all LOD scores over all loci on every chromosome and all available data sets (there were 1917 LOD scores in each genome scan).

VC LOD scores from GENEHUNTER were compared to VC results from MERLIN. For all traits examined the correlation between LOD scores from the two programs was at least 0.993. For 18 of the 23 traits the correlation was 0.9999. All *p*-values were highly significant (*p *< 0.0001).

The glucose A data set, which had the highest kurtosis, was a special problem for regression-based linkage. The program gave no LOD score at all between 180 and 190 cM on chromosome 4, and it gave a LOD score of 12.5 at 1 cM on chromosome 3. In both cases, it gave heritabilities greater than 1. For these reasons, we exclude the glucose A data set from further consideration. In addition, results from MERLIN for weight were obviously incorrect (heritabilities of 1.0 and all LOD scores were 0.0), so we do not report any results for weight. The reasons for this anomaly are currently unknown to us.

Table 3 shows the correlation between pairs of traits for all traits analyzed. The results conform to prior expectation; the highest correlations are within those traits that have the smallest measurement error (height and BMI). Also, the lowest correlations are between the two data sets (A and D) that are the farthest apart in time.

Table 4 shows the MERLIN VC LOD score correlations between all possible pairs of data sets. For cholesterol and HDL, data set C was missing, so correlations are not available for pairs involving data set C. In general, the highest correlations were for BMI and height and the lowest correlations were for glucose and SBP. In addition, there was a crudely inverse relationship between time interval and correlation; i.e., higher correlations were observed when the time interval between exams was smaller (B-C and C-D) and lower correlations were observed when the time interval was larger (A-D).

Table 5 shows the regression-based LOD score correlations between all possible pairs of data sets. The overall pattern among traits is very similar to VC, i.e., higher correlations for BMI and height and lower correlations for glucose and SBP. However, the exam-to-exam consistency is much higher for regression-based linkage, often twice as high.

## Discussion

Two obvious differences between the two methods are the number of significant linkage regions and the improved consistency in LOD scores between data sets provided by the regression-based approach as opposed to the variance components approach. VC analysis found almost three times as many significant regions as regression-based linkage. This may be due to the well known inflation of LOD scores due to non-normality, and indeed two of the significant VC linkages were detected in glucose, which has high kurtosis. However, the other eight significant VC linkages are in traits with low kurtosis (see Table 1). This may indicate that regression-based linkage is conservative. The higher consistency between data sets from the regression-based approach supports the previously reported robustness of this method [2]. It should be noted, however, that the consistency for some traits (e.g., SBP) remains low.

Glucose, which has high kurtosis, is an especially interesting test of the regression method. The VC LOD scores do not appear to be excessive, however, the regression LOD scores are generally lower, overall. The much weaker correlations between exams from VC analysis reconfirms again that VC is sensitive to non-normality. Indeed, given the low trait correlations for glucose (see Table 3), the consistency of the regression method is quite good.

The lack of consistency between exams is somewhat disappointing. Table 2 shows only four regions in which at least three of the four exams had LOD scores greater than 1.44. With the possible exception of height, Tables 4 and 5 confirm the poor consistency between exams. However, the consistency between exams may also be affected by the varying sample size from exam to exam. In addition, the individuals dropping out of the study due to death are more likely to be older. The effect of sample size and dropout bias needs to be investigated further.

The consistency measure used here, correlation between LOD scores, is problematic. The distribution of LOD scores from any two exams is clearly not distributed as a bivariate normal. However, the pattern observed across time, lower correlations across longer time periods, which corresponds to expectations, somewhat ameliorates this concern. Clearly, more work is needed to develop a valid measure of consistency.

The height results are puzzling. Each method found two significant linkages, but neither method gave any support to the significant linkages found by the other method. A closer examination of the height results reveals that this inverse relationship exists for the suggestive linkages as well. The LOD score correlation between methods for height (0.21) is less than half the next lowest (0.51 for SBP). Height is unique in this study in at least two ways, much higher heritability than the other traits and the assortative mating for height in humans. However, it is not clear why either of the two methods should be sensitive to these unique aspects of height. In addition, kurtosis for height is negligible and the highest LOD score from both methods have some support from previously reported genome scans (e.g., Hirschhorn et al. [6]), thus there seems to be little reason to prefer one method to the other.

## Figures and Tables

**Table 1:** Descriptive statistics for all traits

**Trait**	***N***	**Mean**	**SD**	**Range**	**h^2^**	**Skewness**	**Kurtosis**
BMI_A	1931	25.417	4.194	14 – 53	0.348	0.797	1.819
BMI_B	1765	25.950	4.362	15 – 51	0.388	0.932	1.912
BMI_C	1679	26.273	4.605	11 – 49	0.375	0.989	1.993
BMI_D	1680	26.833	4.846	14 – 56	0.458	1.153	2.595
CHOL_A	1319	190.965	37.964	96 – 346	0.616	0.509	0.293
CHOL_B	1779	209.465	41.812	104 – 418	0.480	0.570	0.673
CHOL_D	1666	207.383	39.335	87 – 396	0.382	0.560	0.955
GLU_A	1976	104.482	20.679	64 – 417	0.129	5.340	53.027
GLU_B	1691	96.186	25.788	47 – 369	0.058	5.362	42.922
GLU_C	1656	96.172	31.991	57 – 487	0.202	6.176	49.620
GLU_D	1674	96.884	30.168	42 – 421	0.109	5.237	35.067
HDL_A	1309	50.665	14.200	16 – 118	0.499	0.719	0.905
HDL_B	1760	48.768	13.485	16 – 114	0.468	0.707	0.722
HDL_D	1662	48.973	14.569	19 – 129	0.456	0.876	1.133
HGT_A	1931	65.822	3.953	49 – 78	0.826	0.092	-0.145
HGT_B	1765	65.725	4.077	54 – 78	0.828	0.136	-0.476
HGT_C	1691	65.912	3.968	55 – 78	0.808	0.129	-0.485
HGT_D	1680	65.851	3.985	55 – 78	0.861	0.086	-0.442
LGTG_A	1318	4.311	0.605	2.5 – 6.7	0.434	0.312	0.222
SBP_A	2049	126.276	19.235	78 – 205	0.227	0.937	1.107
SBP_B	1773	125.438	18.300	82 – 203	0.238	0.680	0.548
SBP_C	1729	126.586	19.335	78 – 201	0.282	0.709	0.569
SBP_D	1718	129.219	21.790	80 – 237	0.267	0.871	1.344

**Table 2:** All LOD scores greater than 1.44 (*p *< 0.005) for VC and regression-based (REG) linkage

	**BMI**				**CHOLESTEROL**				**GLUCOSE**	**HEIGHT**				**SBP**			
Chr# /set	cM	VC LOD	REG LOD	Chr# /set	cM	VC LOD	REG LOD	Chr# /set	cM	VC LOD	REG LOD	Chr# /set	cM	VC LOD	REG LOD	Chr# /set	cM	VC LOD	REG LOD

1/C	96.8	1.63	1.06	1/D	75.2	1.14	1.81	1/C	16.0	1.48	0.44	1/A	16.0	2.96	0.03	1/D	192.0	1.48	1.49
2/A	253.8	1.79	1.12	1/A	75.6	2.15	2.09	1/D	196.0	1.12	1.46	1/A	114.0	0.49	2.29	1/D	208.0	2.28	0.57
3/D	170.6	1.67	1.45	1/A	76.0	2.25	2.09	1/D	222.8	1.23	1.80	1/A	265.4	1.78	0.01	2/D	38.0	3.01	0.29
3/D	179.0	1.52	1.10	1/B	76.0	2.51	1.79	1/B	270.8	0.25	1.53	2/C	59.2	1.58	0.10	2/D	87.8	1.59	1.04
3/D	185.6	1.58	0.87	2/B	125.0	1.08	1.55	2/B	12.4	0.37	2.16	2/C	82.2	1.77	-0.01	2/C	126.6	0.42	1.63
3/D	216.0	1.49	1.30	2/B	145.8	1.19	1.53	2/C	114.0	2.34	0.70	3/A	201.0	2.70	0.10	5/C	25.6	1.88	1.72
3/A	225.0	1.38	1.73	5/B	85.0	1.47	1.11	2/D	122.8	1.01	1.47	5/A	43.0	0.38	1.56	5/C	28.2	1.79	1.88
3/D	225.0	1.41	1.59	7/A	51.6	3.46	1.76	2/D	140.2	1.17	2.42	6/A	25.0	4.35	0.08	5/A	40.0	1.63	0.51
4/B	93.0	1.52	1.16	7/D	54.8	2.16	1.18	2/D	148.2	1.27	1.44	6/A	146.0	1.78	0.85	5/C	53.4	1.53	0.30
6/B	139.6	2.62	1.24	7/D	100.2	3.70	3.40	2/C	237.0	1.46	0.81	6/A	153.2	1.11	1.46	6/C	157.2	2.14	0.09
6/C	141.2	2.60	1.73	7/D	104.6	3.98	3.00	3/D	119.0	1.11	1.77	6/A	184.6	3.01	0.56	7/C	152.0	1.51	1.16
6/C	142.8	2.54	1.74	8/B	26.0	1.85	1.10	5/C	23.0	1.80	0.36	7/A	70.0	1.53	0.51	7/C	167.4	1.47	0.65
6/A	146.0	4.97	2.27	11/D	147.0	1.75	2.55	5/C	45.0	1.63	0.25	7/D	182.0	0.37	1.73	7/B	174.0	1.54	0.47
6/A	147.8	5.03	2.23	11/D	148.0	1.75	2.60	5/C	85.0	2.73	0.74	8/B	26.0	0.06	1.99	8/D	37.0	2.37	2.17
8/B	30.4	2.58	0.59	12/B	61.4	0.45	1.89	5/C	175.0	2.11	0.33	8/C	26.0	0.32	2.74	8/D	38.4	2.23	2.17
8/C	32.6	2.09	0.58	15/D	35.8	1.52	1.08	7/C	79.0	3.01	1.07	8/D	26.0	0.31	3.11	8/A	44.0	1.47	0.28
11/B	117.0	3.18	2.60	18/A	16.0	1.34	1.49	7/C	163.0	1.70	0.36	8/B	56.8	0.41	2.30	12/A	32.0	0.15	1.59
11/B	119.0	3.24	2.48	19/D	70.0	2.48	2.10	8/C	1.0	1.45	0.31	9/A	57.2	1.52	1.36	12/A	41.2	0.14	1.63
16/A	45.4	1.13	2.14	19/D	74.0	2.84	1.85	11/C	76.0	2.13	0.41	9/C	58.0	1.62	1.69	12/B	65.8	1.21	3.28
16/B	55.2	1.86	2.40	19/D	82.0	2.49	1.80	18/C	41.0	2.22	0.36	9/A	59.6	1.04	1.45	14/D	56.0	1.49	1.03
16/B	65.6	2.07	2.13	20/D	33.0	1.83	1.93	18/C	89.0	1.80	0.22	9/C	64.4	0.77	2.34	15/C	60.0	0.98	1.70
				20/D	34.2	1.90	1.90	19/C	78.0	2.70	1.70	9/D	80.0	0.97	1.46	15/B	68.0	1.07	1.61
	Corr	0.71979		20/A	35.4	1.51	1.59	19/C	101.0	1.60	0.99	10/D	161.6	0.19	1.49	15/D	122.0	1.30	2.05
				21/B	7.0	1.95	1.47	22/C	46.0	3.99	1.56	12/A	83.0	1.67	0.06	19/B	10.0	1.50	0.79
**HDL**				21/B	11.0	1.72	1.58					12/B	85.4	1.90	0.00	19/D	14.4	1.54	0.63
1/B	8.8	1.91	0.86						Corr	0.53719		13/A	55.0	2.05	0.06	19/D	42.0	1.53	0.26
2/A	131.4	2.09	0.99		Corr	0.79113						14/D	126.0	2.42	0.18	22/B	25.0	0.89	2.38
6/A	136.2	3.00	2.35									17/A	63.0	0.34	2.23				
7/D	51.6	1.76	1.09	**LGTG**								17/B	84.8	0.36	3.22		Corr	0.51023	
7/D	67.6	2.18	1.22	2/A	48.0	1.52	0.73					17/D	86.2	0.11	2.37				
10/B	19.0	1.74	1.30	7/A	67.6	0.83	1.56					17/C	87.6	0.53	4.28				
17/D	120.6	0.18	1.91	18/A	105.4	0.89	1.78					18/A	28.0	2.13	0.39				
19/D	78.0	1.46	1.33	20/A	42.6	3.43	2.30					18/A	98.2	1.75	0.14				
				20/A	44.4	3.58	2.27					19/B	23.4	0.23	2.08				
	Corr	0.64385										19/A	25.8	0.75	1.72				
					Corr	0.71896						20/A	62.0	1.69	0.34				
													Corr	0.21209					

**Table 3:** Correlations of trait values across all pairs of data sets

**Trait**	**A-B**	**B-C**	**C-D**	**A-C**	**B-D**	**A-D**
BMI	0.83118	0.88684	0.89639	0.78071	0.85963	0.74361
CHOL	0.71958	NA	NA	NA	0.67309	0.62801
GLU	0.29413	0.49062	0.55675	0.39816	0.45167	0.35927
HDL	0.67414	NA	NA	NA	0.72461	0.66024
HGT	0.94391	0.96608	0.96617	0.90895	0.97801	0.92227
SBP	0.64297	0.71147	0.74963	0.57700	0.67214	0.52960

**Table 4:** Correlations of MERLIN VC LOD scores across all pairs of data sets

**Trait**	**A-B**	**B-C**	**C-D**	**A-C**	**B-D**	**A-D**
BMI	0.39471	0.58782	0.42459	0.56397	0.44065	0.41956
CHOL	0.34474	NA	NA	NA	0.21560	0.36782
GLU	0.09828	-0.14132	0.19532	0.08657	0.17764	-0.19882
HDL	0.03541	NA	NA	NA	0.17621	0.14310
HGT	0.24197	0.53309	0.49156	0.11526	0.53223	0.25013
SBP	0.11614	0.29417	0.32525	0.13484	0.39478	0.10886

**Table 5:** Correlations of MERLIN regression-based LOD scores across all pairs of data sets

**Trait**	**A-B**	**B-C**	**C-D**	**A-C**	**B-D**	**A-D**
BMI	0.53675	0.53764	0.59845	0.50334	0.53153	0.50164
CHOL	0.36095	NA	NA	NA	0.31658	0.34541
GLU	NA	0.16786	0.39032	NA	0.33118	NA
HDL	0.25531	NA	NA	NA	0.36504	0.20726
HGT	0.63828	0.78861	0.81809	0.68092	0.81538	0.68434
SBP	0.41234	0.41590	0.47115	0.25181	0.39897	0.13721
